# Supplementary material for: Single Cell Transcriptomic and Chromatin Profiles Suggest Layer Vb Is the Only Layer With Shared Excitatory Cell Types in the Medial and Lateral Entorhinal Cortex
Source: Front Neural Circuits. 2022 Jan 26;15:806154. doi: 10.3389/fncir.2021.806154 (PMC8826650; doi:10.3389/fncir.2021.806154)
Supplement: Supplementary file 14 [file Data_Sheet_1.ZIP › 1/knownResults.html]

1 - Homer Known Motif Enrichment Results


# Homer Known Motif Enrichment Results (1)

Homer *de novo* Motif Results  
Gene Ontology Enrichment Results  
Known Motif Enrichment Results (txt file)  
Total Target Sequences = 1242, Total Background Sequences = 47891

|  |  |  |  |  |  |  |  |  |  |  |  |
| --- | --- | --- | --- | --- | --- | --- | --- | --- | --- | --- | --- |
| Rank | Motif | Name | P-value | log P-pvalue | q-value (Benjamini) | # Target Sequences with Motif | % of Targets Sequences with Motif | # Background Sequences with Motif | % of Background Sequences with Motif | Motif File | SVG |
| 1 | C T A G T C G A T G A C A G T C C G T A A C T G G T A C A C G T A C T G A C T G | BHLHA15(bHLH)/NIH3T3-BHLHB8.HA-ChIP-Seq(GSE119782)/Homer | 1e-106 | -2.448e+02 | 0.0000 | 596.0 | 47.99% | 9626.0 | 20.10% | motif file (matrix) | svg |
| 2 | T G A C A G T C C G T A A C T G G T A C A C G T A C T G A C G T G A C T G A T C | Twist2(bHLH)/Myoblast-Twist2.Ty1-ChIP-Seq(GSE127998)/Homer | 1e-96 | -2.225e+02 | 0.0000 | 676.0 | 54.43% | 12608.1 | 26.33% | motif file (matrix) | svg |
| 3 | T A C G T G C A A G T C C G T A A C G T T G A C A C G T A C T G A C T G G C A T | TCF4(bHLH)/SHSY5Y-TCF4-ChIP-Seq(GSE96915)/Homer | 1e-96 | -2.223e+02 | 0.0000 | 626.0 | 50.40% | 11048.8 | 23.07% | motif file (matrix) | svg |
| 4 | T C G A T G A C G T A C C G T A C A G T T G A C A C G T A C T G A G C T A G C T | NeuroG2(bHLH)/Fibroblast-NeuroG2-ChIP-Seq(GSE75910)/Homer | 1e-95 | -2.199e+02 | 0.0000 | 617.0 | 49.68% | 10832.6 | 22.62% | motif file (matrix) | svg |
| 5 | T C A G A C G T T C G A T A G C A G T C C G T A A C T G G T A C A C G T A C T G A T C G A G T C | Atoh1(bHLH)/Cerebellum-Atoh1-ChIP-Seq(GSE22111)/Homer | 1e-88 | -2.036e+02 | 0.0000 | 466.0 | 37.52% | 6932.3 | 14.48% | motif file (matrix) | svg |
| 6 | C T G A T G A C T G A C C G T A A C G T T G A C A G C T C T A G A C G T G A C T | Olig2(bHLH)/Neuron-Olig2-ChIP-Seq(GSE30882)/Homer | 1e-87 | -2.013e+02 | 0.0000 | 712.0 | 57.33% | 14393.7 | 30.06% | motif file (matrix) | svg |
| 7 | T C A G T G A C G T A C C G T A A C G T T G A C A C G T T C A G A G C T G A C T | NeuroD1(bHLH)/Islet-NeuroD1-ChIP-Seq(GSE30298)/Homer | 1e-70 | -1.626e+02 | 0.0000 | 365.0 | 29.39% | 5196.5 | 10.85% | motif file (matrix) | svg |
| 8 | C G A T T G C A T G C A G A T C C G T A A C T G T G A C G A C T C A T G A C T G | Tcf21(bHLH)/ArterySmoothMuscle-Tcf21-ChIP-Seq(GSE61369)/Homer | 1e-38 | -8.974e+01 | 0.0000 | 313.0 | 25.20% | 5586.8 | 11.67% | motif file (matrix) | svg |
| 9 | C T A G A G T C T A C G T A C G T G A C C G T A A C T G T A G C G C A T C A T G A T G C A G C T | Ascl1(bHLH)/NeuralTubes-Ascl1-ChIP-Seq(GSE55840)/Homer | 1e-38 | -8.873e+01 | 0.0000 | 426.0 | 34.30% | 8918.1 | 18.62% | motif file (matrix) | svg |
| 10 | C T G A T C A G G T A C G C T A A C T G T G A C G C A T C A T G | SCL(bHLH)/HPC7-Scl-ChIP-Seq(GSE13511)/Homer | 1e-36 | -8.508e+01 | 0.0000 | 934.0 | 75.20% | 27681.9 | 57.81% | motif file (matrix) | svg |
| 11 | T C G A A G T C C G T A A T C G T A G C A C G T A C T G A G C T A C G T A G T C | Ptf1a(bHLH)/Panc1-Ptf1a-ChIP-Seq(GSE47459)/Homer | 1e-30 | -7.125e+01 | 0.0000 | 624.0 | 50.24% | 16331.3 | 34.10% | motif file (matrix) | svg |
| 12 | A G C T A C G T A C T G A T G C A G T C C G T A C T G A T A C G | NF1-halfsite(CTF)/LNCaP-NF1-ChIP-Seq(Unpublished)/Homer | 1e-29 | -6.689e+01 | 0.0000 | 450.0 | 36.23% | 10588.3 | 22.11% | motif file (matrix) | svg |
| 13 | G T C A T G C A G C T A A G T C C G T A A C T G T G A C G C A T T C A G C A G T | Ap4(bHLH)/AML-Tfap4-ChIP-Seq(GSE45738)/Homer | 1e-24 | -5.570e+01 | 0.0000 | 333.0 | 26.81% | 7384.6 | 15.42% | motif file (matrix) | svg |
| 14 | C T G A C T A G A C T G G C A T C T A G G C A T A T C G C T G A C G T A G T C A | Tbx21(T-box)/GM12878-TBX21-ChIP-Seq(Encode)/Homer | 1e-21 | -4.916e+01 | 0.0000 | 299.0 | 24.07% | 6630.7 | 13.85% | motif file (matrix) | svg |
| 15 | T G A C A T G C C G T A A T C G A T G C C A G T C A T G A C T G A G T C G T A C | HEB(bHLH)/mES-Heb-ChIP-Seq(GSE53233)/Homer | 1e-17 | -4.103e+01 | 0.0000 | 416.0 | 33.49% | 10835.4 | 22.63% | motif file (matrix) | svg |
| 16 | T G C A C T G A A T G C G T C A A C G T A T G C A C G T A C T G A C T G T G C A | ZBTB18(Zf)/HEK293-ZBTB18.GFP-ChIP-Seq(GSE58341)/Homer | 1e-17 | -4.069e+01 | 0.0000 | 171.0 | 13.77% | 3240.1 | 6.77% | motif file (matrix) | svg |
| 17 | T C G A A G T C C G T A A T C G A T G C C G A T A C T G A G T C A G C T A C T G | Tcf12(bHLH)/GM12878-Tcf12-ChIP-Seq(GSE32465)/Homer | 1e-16 | -3.860e+01 | 0.0000 | 232.0 | 18.68% | 5076.1 | 10.60% | motif file (matrix) | svg |
| 18 | G T A C G A T C C A G T A G T C A G T C A G T C T G C A G A T C C T G A A T G C G T C A A C G T | WT1(Zf)/Kidney-WT1-ChIP-Seq(GSE90016)/Homer | 1e-16 | -3.709e+01 | 0.0000 | 129.0 | 10.39% | 2235.9 | 4.67% | motif file (matrix) | svg |
| 19 | G C T A G A C T G A C T T G C A C G T A A G T C C G T A T A G C G A T C G A C T | Eomes(T-box)/H9-Eomes-ChIP-Seq(GSE26097)/Homer | 1e-16 | -3.698e+01 | 0.0000 | 539.0 | 43.40% | 15405.7 | 32.17% | motif file (matrix) | svg |
| 20 | T C G A T C G A A G T C C G T A C T A G T A G C A C G T A C T G | MyoG(bHLH)/C2C12-MyoG-ChIP-Seq(GSE36024)/Homer | 1e-15 | -3.649e+01 | 0.0000 | 252.0 | 20.29% | 5782.9 | 12.08% | motif file (matrix) | svg |
| 21 | A C G T T C G A T C G A A G T C G T C A T A C G A T G C A C G T A C T G A G C T | Myf5(bHLH)/GM-Myf5-ChIP-Seq(GSE24852)/Homer | 1e-14 | -3.397e+01 | 0.0000 | 186.0 | 14.98% | 3921.0 | 8.19% | motif file (matrix) | svg |
| 22 | G A C T C T A G A T G C A G T C G T C A T A C G A T G C A T C G | HIC1(Zf)/Treg-ZBTB29-ChIP-Seq(GSE99889)/Homer | 1e-14 | -3.283e+01 | 0.0000 | 420.0 | 33.82% | 11513.6 | 24.04% | motif file (matrix) | svg |
| 23 | C T G A C T G A C A T G A T C G A G C T A T C G G A C T C A T G C T G A G T C A | Tbr1(T-box)/Cortex-Tbr1-ChIP-Seq(GSE71384)/Homer | 1e-14 | -3.252e+01 | 0.0000 | 379.0 | 30.52% | 10133.8 | 21.16% | motif file (matrix) | svg |
| 24 | C G T A C T A G C A T G A G C T A C T G C G A T A T C G C G T A G T C A G T C A | Tbet(T-box)/CD8-Tbet-ChIP-Seq(GSE33802)/Homer | 1e-12 | -2.868e+01 | 0.0000 | 284.0 | 22.87% | 7230.3 | 15.10% | motif file (matrix) | svg |
| 25 | T C G A A C G T A C G T C T G A G A T C T C A G G A C T G T C A C G T A A G C T G T C A C T A G A G C T A C G T T C G A | NFIL3(bZIP)/HepG2-NFIL3-ChIP-Seq(Encode)/Homer | 1e-11 | -2.672e+01 | 0.0000 | 213.0 | 17.15% | 5084.8 | 10.62% | motif file (matrix) | svg |
| 26 | G A C T C G A T C T G A G T C A G A C T C G A T T C G A C G T A G C T A G C T A T G A C G T A C C G T A A C T G T G C A C G A T A C T G A C G T | Pitx1:Ebox(Homeobox,bHLH)/Hindlimb-Pitx1-ChIP-Seq(GSE41591)/Homer | 1e-10 | -2.470e+01 | 0.0000 | 91.0 | 7.33% | 1627.9 | 3.40% | motif file (matrix) | svg |
| 27 | C T G A T C A G A G T C C G T A A T C G A T G C C G A T A C T G A G T C G A C T A T C G A G T C | MyoD(bHLH)/Myotube-MyoD-ChIP-Seq(GSE21614)/Homer | 1e-10 | -2.412e+01 | 0.0000 | 175.0 | 14.09% | 4053.5 | 8.46% | motif file (matrix) | svg |
| 28 | C T A G C T G A T C A G A T C G A G C T C A T G G A C T A G T C C T G A T G C A | Tbx6(T-box)/ESC-Tbx6-ChIP-Seq(GSE93524)/Homer | 1e-8 | -1.952e+01 | 0.0000 | 249.0 | 20.05% | 6706.4 | 14.00% | motif file (matrix) | svg |
| 29 | T G A C G T A C C G T A A C T G T G A C C G A T A C T G A T C G A G C T T A C G T C G A T A G C G T A C C G T A A T C G T G A C G C A T A C T G A C T G A T G C | Twist(bHLH)/HMLE-TWIST1-ChIP-Seq(Chang\_et\_al)/Homer | 1e-8 | -1.932e+01 | 0.0000 | 48.0 | 3.86% | 707.0 | 1.48% | motif file (matrix) | svg |
| 30 | T C A G G A C T C A G T C T G A A G C T C T A G G A C T T G C A C T G A A G T C | HLF(bZIP)/HSC-HLF.Flag-ChIP-Seq(GSE69817)/Homer | 1e-7 | -1.687e+01 | 0.0000 | 257.0 | 20.69% | 7184.6 | 15.00% | motif file (matrix) | svg |
| 31 | C G T A T G A C T C G A A G T C C G T A A T C G A T G C A C G T A C T G A G T C | E2A(bHLH)/proBcell-E2A-ChIP-Seq(GSE21978)/Homer | 1e-6 | -1.521e+01 | 0.0000 | 273.0 | 21.98% | 7876.7 | 16.45% | motif file (matrix) | svg |
| 32 | T A G C G T A C C G T A C T A G A C T G T G C A C G T A A T G C C G T A A T C G | AR-halfsite(NR)/LNCaP-AR-ChIP-Seq(GSE27824)/Homer | 1e-6 | -1.510e+01 | 0.0000 | 746.0 | 60.06% | 25365.0 | 52.97% | motif file (matrix) | svg |
| 33 | G A C T C A G T A G C T C G A T A G T C G A T C A G T C C G T A A T G C T C A G | Rbpj1(?)/Panc1-Rbpj1-ChIP-Seq(GSE47459)/Homer | 1e-6 | -1.455e+01 | 0.0000 | 326.0 | 26.25% | 9784.7 | 20.43% | motif file (matrix) | svg |
| 34 | T A C G T A C G C T A G T C A G A G T C C G T A A T C G A T G C A C G T A C T G A G T C G A C T | Ascl2(bHLH)/ESC-Ascl2-ChIP-Seq(GSE97712)/Homer | 1e-5 | -1.286e+01 | 0.0000 | 211.0 | 16.99% | 5979.0 | 12.49% | motif file (matrix) | svg |
| 35 | C A G T T C A G G A T C A C T G A C G T C T A G A C T G A C T G G A C T C T A G | Egr1(Zf)/K562-Egr1-ChIP-Seq(GSE32465)/Homer | 1e-5 | -1.247e+01 | 0.0000 | 110.0 | 8.86% | 2714.1 | 5.67% | motif file (matrix) | svg |
| 36 | T G C A A G C T A C G T C T A G G A T C C T A G G A T C G T C A C T G A A G T C | CEBP(bZIP)/ThioMac-CEBPb-ChIP-Seq(GSE21512)/Homer | 1e-4 | -1.090e+01 | 0.0002 | 176.0 | 14.17% | 4978.5 | 10.40% | motif file (matrix) | svg |
| 37 | A G C T C A T G G C A T G A T C T G C A C T A G G A T C A C G T | Tgif2(Homeobox)/mES-Tgif2-ChIP-Seq(GSE55404)/Homer | 1e-4 | -9.890e+00 | 0.0006 | 640.0 | 51.53% | 22021.0 | 45.98% | motif file (matrix) | svg |
| 38 | C T G A C T A G A T C G A G C T A C T G G A C T A G T C C T G A | Tbx5(T-box)/HL1-Tbx5.biotin-ChIP-Seq(GSE21529)/Homer | 1e-4 | -9.533e+00 | 0.0008 | 575.0 | 46.30% | 19599.7 | 40.93% | motif file (matrix) | svg |
| 39 | T A C G A T G C G C T A A C T G C G T A A C G T C G T A C T G A T A C G T C G A | Gata4(Zf)/Heart-Gata4-ChIP-Seq(GSE35151)/Homer | 1e-3 | -9.003e+00 | 0.0014 | 261.0 | 21.01% | 8124.0 | 16.96% | motif file (matrix) | svg |
| 40 | A G C T A G T C A T G C A G C T A C G T C G T A A C G T A G T C C G A T A T G C | Gata2(Zf)/K562-GATA2-ChIP-Seq(GSE18829)/Homer | 1e-3 | -8.811e+00 | 0.0016 | 171.0 | 13.77% | 5011.4 | 10.46% | motif file (matrix) | svg |
| 41 | T C A G G C T A T C A G C A G T T G A C G T C A A G T C A T C G T G C A G T A C C A G T G A T C | Npas4(bHLH)/Neuron-Npas4-ChIP-Seq(GSE127793)/Homer | 1e-3 | -8.515e+00 | 0.0022 | 126.0 | 10.14% | 3523.5 | 7.36% | motif file (matrix) | svg |
| 42 | A G T C G A C T C A G T A C T G C T A G T G A C G C T A A T G C G C A T A T C G C G A T A C T G G A T C G T A C G T C A C T G A | NF1(CTF)/LNCAP-NF1-ChIP-Seq(Unpublished)/Homer | 1e-3 | -8.343e+00 | 0.0025 | 74.0 | 5.96% | 1855.5 | 3.87% | motif file (matrix) | svg |
| 43 | G C T A A T C G G C T A G A C T G C T A T C G A T A G C T C G A | GATA3(Zf)/iTreg-Gata3-ChIP-Seq(GSE20898)/Homer | 1e-3 | -8.013e+00 | 0.0034 | 382.0 | 30.76% | 12640.2 | 26.40% | motif file (matrix) | svg |
| 44 | T A C G T A C G G T A C A T C G A C T G T A C G T C G A C T G A T C G A A T C G | E2F6(E2F)/Hela-E2F6-ChIP-Seq(GSE31477)/Homer | 1e-3 | -7.927e+00 | 0.0036 | 53.0 | 4.27% | 1236.5 | 2.58% | motif file (matrix) | svg |
| 45 | T C A G A G C T A C G T A C G T G T A C G A T C C G T A C T A G C A T G G T C A C G T A T C G A | STAT4(Stat)/CD4-Stat4-ChIP-Seq(GSE22104)/Homer | 1e-3 | -7.543e+00 | 0.0052 | 229.0 | 18.44% | 7180.8 | 14.99% | motif file (matrix) | svg |
| 46 | C A G T T G C A A C G T A C T G C G T A A T C G C G A T T G A C C G T A A C G T | BATF(bZIP)/Th17-BATF-ChIP-Seq(GSE39756)/Homer | 1e-3 | -7.305e+00 | 0.0064 | 141.0 | 11.35% | 4145.4 | 8.66% | motif file (matrix) | svg |
| 47 | T G C A A G C T C A T G C G T A A G C T A C T G G A T C G T C A C G T A A G C T | Atf4(bZIP)/MEF-Atf4-ChIP-Seq(GSE35681)/Homer | 1e-3 | -7.113e+00 | 0.0076 | 77.0 | 6.20% | 2036.8 | 4.25% | motif file (matrix) | svg |
| 48 | C G T A C G A T G A C T G A C T T G A C C T G A A T G C C T G A T A G C A G T C A C G T T C G A C A T G T A C G G A C T A T C G G A C T A C G T C T G A T C G A C G T A | Brachyury(T-box)/Mesoendoderm-Brachyury-ChIP-exo(GSE54963)/Homer | 1e-3 | -7.102e+00 | 0.0076 | 71.0 | 5.72% | 1845.6 | 3.85% | motif file (matrix) | svg |
| 49 | A C T G C T A G T C G A C G A T C A T G G C T A A T C G C G A T G T A C G C T A A G C T G T A C | Fra1(bZIP)/BT549-Fra1-ChIP-Seq(GSE46166)/Homer | 1e-3 | -6.993e+00 | 0.0082 | 120.0 | 9.66% | 3465.0 | 7.24% | motif file (matrix) | svg |
| 50 | A G C T A T G C G A C T G C A T C G T A A G C T G T A C C G A T A T C G A G T C | Gata6(Zf)/HUG1N-GATA6-ChIP-Seq(GSE51936)/Homer | 1e-3 | -6.980e+00 | 0.0082 | 234.0 | 18.84% | 7435.7 | 15.53% | motif file (matrix) | svg |
| 51 | A G T C G A C T C A G T G T A C A G T C A T C G T C A G A C T G G T C A C G T A | Stat3(Stat)/mES-Stat3-ChIP-Seq(GSE11431)/Homer | 1e-3 | -6.977e+00 | 0.0082 | 108.0 | 8.70% | 3064.4 | 6.40% | motif file (matrix) | svg |
| 52 | T G A C C T G A C T A G C T G A C G T A A G T C C T G A A C G T G C A T T A G C G C A T A T C G G A C T G A C T G A T C | GRE(NR),IR3/RAW264.7-GRE-ChIP-Seq(Unpublished)/Homer | 1e-3 | -6.923e+00 | 0.0083 | 58.0 | 4.67% | 1448.8 | 3.03% | motif file (matrix) | svg |
| 53 | T A G C T C A G C A T G G C A T A G C T C G A T A T G C C G T A C G T A G T C A | CHR(?)/Hela-CellCycle-Expression/Homer | 1e-2 | -6.509e+00 | 0.0124 | 174.0 | 14.01% | 5378.7 | 11.23% | motif file (matrix) | svg |
| 54 | G A C T C G A T T C A G G A T C G A C T A G C T A G C T A G T C G A T C C G T A C T A G C T A G T C G A T C G A C T G A | Bcl6(Zf)/Liver-Bcl6-ChIP-Seq(GSE31578)/Homer | 1e-2 | -6.415e+00 | 0.0133 | 318.0 | 25.60% | 10559.5 | 22.05% | motif file (matrix) | svg |
| 55 | C A T G A C T G C T A G T C G A T C G A T C G A T C G A T C A G T C A G T C A G T G A C T G A C C G T A A C T G T G C A C G A T A C T G | RBPJ:Ebox(?,bHLH)/Panc1-Rbpj1-ChIP-Seq(GSE47459)/Homer | 1e-2 | -6.414e+00 | 0.0133 | 76.0 | 6.12% | 2058.2 | 4.30% | motif file (matrix) | svg |
| 56 | A G T C A T C G C T A G A G C T G A C T C T A G A G T C A G T C G C T A C A G T T C A G T C A G G A T C C T G A T C G A G A T C | RFX(HTH)/K562-RFX3-ChIP-Seq(SRA012198)/Homer | 1e-2 | -6.380e+00 | 0.0133 | 20.0 | 1.61% | 363.4 | 0.76% | motif file (matrix) | svg |
| 57 | C A T G C T A G A G C T G A C T C A T G A G T C G A T C G C T A C G A T C T A G T C A G G T A C C T G A T C G A | X-box(HTH)/NPC-H3K4me1-ChIP-Seq(GSE16256)/Homer | 1e-2 | -6.341e+00 | 0.0136 | 30.0 | 2.42% | 639.1 | 1.33% | motif file (matrix) | svg |
| 58 | A C G T C T A G C G T A A G T C G T A C A C G T A C G T A C G T G T C A G T A C T G A C G A C T | Nur77(NR)/K562-NR4A1-ChIP-Seq(GSE31363)/Homer | 1e-2 | -6.239e+00 | 0.0148 | 49.0 | 3.95% | 1212.8 | 2.53% | motif file (matrix) | svg |
| 59 | A C T G C A T G T C G A A C G T A C T G C G T A A T C G A C G T G T A C C G T A G A C T A G T C | Fos(bZIP)/TSC-Fos-ChIP-Seq(GSE110950)/Homer | 1e-2 | -6.215e+00 | 0.0149 | 125.0 | 10.06% | 3716.5 | 7.76% | motif file (matrix) | svg |
| 60 | A T G C T A G C A G C T A G C T T G A C G A C T T C A G T A C G G T C A C T G A A T C G T A G C G A C T C A G T A G T C A G C T T C G A A T C G T G C A T G C A | HRE(HSF)/HepG2-HSF1-ChIP-Seq(GSE31477)/Homer | 1e-2 | -6.181e+00 | 0.0152 | 42.0 | 3.38% | 1001.9 | 2.09% | motif file (matrix) | svg |
| 61 | T G A C G C T A T G A C C G T A T C A G G A T C C G T A C A T G C A T G C T A G C T A G C T A G | Unknown-ESC-element(?)/mES-Nanog-ChIP-Seq(GSE11724)/Homer | 1e-2 | -6.115e+00 | 0.0159 | 99.0 | 7.97% | 2845.0 | 5.94% | motif file (matrix) | svg |
| 62 | C T G A C G A T C T A G T C A G G A T C C T G A T C A G G A T C C T G A A C T G A G T C G C T A A C G T A G T C G C A T | PRDM9(Zf)/Testis-DMC1-ChIP-Seq(GSE35498)/Homer | 1e-2 | -6.105e+00 | 0.0159 | 89.0 | 7.17% | 2512.5 | 5.25% | motif file (matrix) | svg |
| 63 | T A G C G C T A A C T G C G T A A C G T C G T A C G T A T A C G T C A G T C G A | Gata1(Zf)/K562-GATA1-ChIP-Seq(GSE18829)/Homer | 1e-2 | -5.976e+00 | 0.0177 | 143.0 | 11.51% | 4364.4 | 9.11% | motif file (matrix) | svg |
| 64 | C T A G T C G A A C G T A C T G C G T A A T G C A C G T G T A C C G T A A G C T G A T C G T A C | Atf3(bZIP)/GBM-ATF3-ChIP-Seq(GSE33912)/Homer | 1e-2 | -5.850e+00 | 0.0198 | 135.0 | 10.87% | 4102.8 | 8.57% | motif file (matrix) | svg |
| 65 | C G T A A C T G C G T A A C G T C G T A C T G A T C A G T G C A A G C T T G A C | TRPS1(Zf)/MCF7-TRPS1-ChIP-Seq(GSE107013)/Homer | 1e-2 | -5.781e+00 | 0.0209 | 494.0 | 39.77% | 17231.3 | 35.98% | motif file (matrix) | svg |
| 66 | C T G A A G C T A C G T A C G T A G T C G A C T G A C T C T G A C T G A C T A G C G T A C G T A | STAT6(Stat)/CD4-Stat6-ChIP-Seq(GSE22104)/Homer | 1e-2 | -5.517e+00 | 0.0268 | 138.0 | 11.11% | 4247.5 | 8.87% | motif file (matrix) | svg |
| 67 | C G A T A C G T A C G T A C G T C G T A A G C T C A G T C T A G A T C G A C T G | HOXB13(Homeobox)/ProstateTumor-HOXB13-ChIP-Seq(GSE56288)/Homer | 1e-2 | -5.498e+00 | 0.0269 | 291.0 | 23.43% | 9733.5 | 20.33% | motif file (matrix) | svg |
| 68 | C T G A A C G T A C G T A C G T A G T C G A C T C G A T C T G A A C T G C G T A C G T A T C G A | STAT5(Stat)/mCD4+-Stat5-ChIP-Seq(GSE12346)/Homer | 1e-2 | -5.081e+00 | 0.0402 | 90.0 | 7.25% | 2646.8 | 5.53% | motif file (matrix) | svg |
| 69 | G A C T C A G T G A T C G A T C A C G T G A T C C T G A T A C G C G T A G T C A | STAT6(Stat)/Macrophage-Stat6-ChIP-Seq(GSE38377)/Homer | 1e-2 | -5.032e+00 | 0.0416 | 137.0 | 11.03% | 4275.9 | 8.93% | motif file (matrix) | svg |
| 70 | A G T C C G A T A C T G A T C G T G A C G C T A C A T G A T C G T G A C C G A T A C T G T A G C G T A C G T C A | Tlx?(NR)/NPC-H3K4me1-ChIP-Seq(GSE16256)/Homer | 1e-2 | -4.950e+00 | 0.0445 | 82.0 | 6.60% | 2389.6 | 4.99% | motif file (matrix) | svg |
| 71 | T C A G T C A G G C T A C G T A T A C G G A C T T C A G T C G A C T G A C G T A T A C G G A C T | IRF8(IRF)/BMDM-IRF8-ChIP-Seq(GSE77884)/Homer | 1e-2 | -4.801e+00 | 0.0509 | 84.0 | 6.76% | 2472.1 | 5.16% | motif file (matrix) | svg |
| 72 | A G C T G C A T A C T G A C G T A G T C A C G T C T A G T A C G | Smad3(MAD)/NPC-Smad3-ChIP-Seq(GSE36673)/Homer | 1e-2 | -4.773e+00 | 0.0517 | 534.0 | 43.00% | 18977.6 | 39.63% | motif file (matrix) | svg |
